# Dissociation of Connectivity for Syntactic Irregularity and Perceptual Ambiguity in Musical Chord Stimuli

**DOI:** 10.3389/fnins.2021.693629

**Published:** 2021-08-30

**Authors:** Chan Hee Kim, Seung-Hyun Jin, June Sic Kim, Youn Kim, Suk Won Yi, Chun Kee Chung

**Affiliations:** ^1^Interdisciplinary Program in Neuroscience, College of Natural Science, Seoul National University, Seoul, South Korea; ^2^Department of Neurosurgery, MEG Center, Seoul National University Hospital, Seoul, South Korea; ^3^Research Institute of Basic Sciences, Seoul National University, Seoul, South Korea; ^4^Department of Music, School of Humanities, The University of Hong Kong, Hong Kong, Hong Kong SAR China; ^5^College of Music, Seoul National University, Seoul, South Korea; ^6^Western Music Research Institute, Seoul National University, Seoul, South Korea; ^7^Department of Brain and Cognitive Science, College of Natural Science, Seoul National University, Seoul, South Korea; ^8^Department of Neurosurgery, Seoul National University Hospital, Seoul, South Korea

**Keywords:** syntactic irregularity, perceptual ambiguity, effective connectivity, linearized time-delayed mutual information, inferior frontal gyrus, superior temporal gyrus, magnetoencephalography

## Abstract

Musical syntax has been studied mainly in terms of “syntactic irregularity” in harmonic/melodic sequences. However, “perceptual ambiguity” referring to the uncertainty of judgment/classification of presented stimuli can in addition be involved in our musical stimuli using three different chord sequences. The present study addresses how “syntactic irregularity” and “perceptual ambiguity” on musical syntax are dissociated, in terms of effective connectivity between the bilateral inferior frontal gyrus (IFGs) and superior temporal gyrus (STGs) by linearized time-delayed mutual information (LTDMI). Three conditions were of five-chord sequences with endings of dominant to tonic, dominant to submediant, and dominant to supertonic. The dominant to supertonic is most irregular, compared with the regular dominant to tonic. The dominant to submediant of the less irregular condition is the most ambiguous condition. In the LTDMI results, connectivity from the right to the left IFG (IFG-LTDMI) was enhanced for the most irregular condition, whereas that from the right to the left STG (STG-LTDMI) was enhanced for the most ambiguous condition (*p* = 0.024 in IFG-LTDMI, *p* < 0.001 in STG-LTDMI, false discovery rate (FDR) corrected). Correct rate was negatively correlated with STG-LTDMI, further reflecting perceptual ambiguity (*p* = 0.026). We found for the first time that syntactic irregularity and perceptual ambiguity coexist in chord stimulus testing musical syntax and that the two processes are dissociated in interhemispheric connectivities in the IFG and STG, respectively.

## Introduction

Early right anterior negativity (ERAN) appearing with the negative peak at about 100–200 ms from stimulus onset reflects the degree of expectation violations in chordal and melodic sequences; i.e., the more a chord and a melody are unexpected, and the more the peak amplitude of the ERAN increases (Koelsch et al., 2000; Koelsch and Jentschke, 2010). The inferior frontal gyrus (IFG) is well known as the generator of ERAN (Maess et al., 2001). The bilateral IFGs play crucial roles in the processing of harmonic expectation (Sammler et al., 2011), though the right IFG shows hemispheric dominance in syntactic irregularity processing (Koelsch et al., 2002). The ERAN is observed in both musicians and non-musicians alike (Koelsch et al., 2000, 2001; Maess et al., 2001), even though it is influenced by musical expertise (Koelsch et al., 2002).

In our previous magnetoencephalography (MEG) study on the ERAN (Kim et al., 2011), using three different stimuli, which consisted of three levels of “regular” (dominant to tonic), “less irregular” (dominant to submediant), and “irregular” (dominant to supertonic) conditions on the conditional probability, the ERAN was not observed in the less irregular condition but in the irregular condition. In the present study, we reanalyzed our previous MEG data on the ERAN, using linearized time-delayed mutual information (LTDMI; see section “Materials and Methods”). The LTDMI is an effective connectivity measure that can calculate information transmission between two time series by giving time delays, which is an ideal measure to test the hypothesis of the present study focusing on the verification of information transmission between specific brain regions based on the time domain. Our hypothesis is first that the connectivity between the bilateral IFGs would be increased with the “dominant to supertonic,” eliciting the largest ERAN. Secondly, we hypothesized that perceptual ambiguity could be separately processed with syntactic irregularity. Through the questionnaire after the experiment on the participants, we confirmed the report that they (66.6% of respondents) felt the most uncertain when discriminating “dominant to submediant.” The term “ambiguity” generally refers to uncertainty caused by two or more plausible interpretations of an object (Tuggy, 1993; Kennedy, 2019). In the present study, perceptual ambiguity refers to the uncertainty of judgment/classification of presented stimuli. Therefore, the “dominant to submediant” of the less irregular condition might elicit a neural response to perceptual ambiguity in areas other than the IFG, instead of the ERAN. Additionally, we tested whether the LTDMI reflecting perceptual ambiguity would be correlated with correct rate (CR) in the behavioral experiment, since the more ambiguous it is to distinguish the conditions, and the lower CR will be.

In terms of statistical learning, both musicians and non-musicians alike who have been exposed to a system of Western tonal music could react sensitively to conditional probability on chord progression in the Bach Choral (Rohrmeier and Cross, 2008). Therefore, if the ability to detect perceptual ambiguity is a basic musical ability, both musicians and non-musicians can react to perceptual ambiguity, and like the ability to detect syntactic irregularity reflected in ERAN.

## Materials and Methods

### Ethics Statement

In the present study, we used the same data sets of our previous study (Kim et al., 2011) that was approved by the Institutional Review Board of the Clinical Research Institute, Seoul National University Hospital (H-1001-020-306); but in order to apply a novel hypothesis, the entire process was newly analyzed based on the analysis procedure of Kim et al. (2011). We reanalyzed the data set to apply novel hypotheses and analyses. All participants provided informed consent of written form prior to the experiments.

### Participants

All 19 participants were women (mean age, 24.3 ± 3.0 years): 9 were musicians and 10 were non-musicians. Musicians (nine piano majors and one violin major) majored in musical instruments at the College of Music and received training from the age of 5 for at least 15 years. Most of the non-musicians had experience taking piano lessons, but they were not music majors. Considering the difference in hemispheric dominance of syntactic processing according to gender, only women were recruited (Koelsch et al., 2003; Kim et al., 2011). They all had normal hearing and were right-handed.

### Musical Stimuli

**FIGURE 1:**
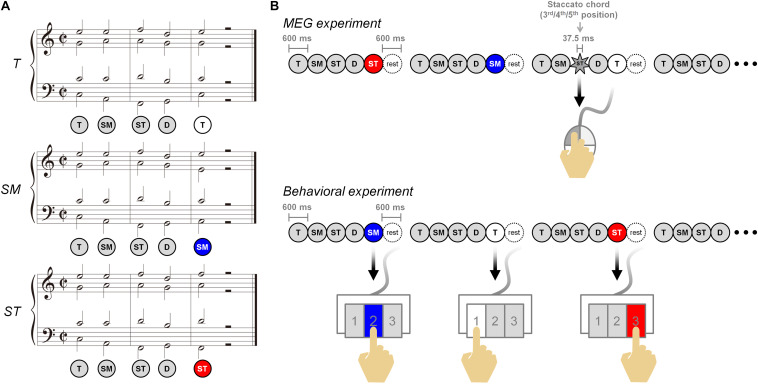
Musical stimuli and experimental paradigm. **(A)** Musical stimuli are composed of three different five-chord sequences. The three conditions are *T*, *SM*, and *ST*, ending with dominant to tonic, dominant to submediant, and dominant to supertonic, respectively. **(B)** In the magnetoencephalography experiment (top), the participants listened to the three conditions carefully and were asked to detect the staccato chord among chord sequences in the three conditions, and to click a mouse (to check the level of attending the conditions). In the behavioral experiment (bottom), the participants discriminated the three conditions and responded by using the 1, 2, and 3 buttons on the keypad.

We used three different conditions: *T*, *SM*, and *ST* (*T* = most regular; *SM* = less regular; *ST* = most irregular; and see also Figure 1A). The *T* was composed of “tonic–submediant–supertonic–dominant–tonic.” The ending chord of “tonic” was replaced with “submediant” and “supertonic” in the *SM* and *ST*, respectively. The chords from the first to fourth were the same in all conditions. In each condition, the duration of a chord was 600 ms. A chord sequence totaled 3,600 ms, including five chords and a 600-ms resting period (Figure 1B). All conditions transposed into 12 major keys, were randomly shuffled in each session, and were recorded at 100 BPM using Cubase 5 (Steinberg Media Technologies, Hamburg, Germany) software. The intensity was normalized in each wave file (sampling rate 44.1 kHz; 16-bit; stereo; windows PCM) using Cool Edit Pro 2.1 (Syntrillium Software Corporation, Phoenix, AZ, United States). The piano timbre (Bösendorfer 290 Imperial grand) in each chord was created by Grand 3 (Steinberg Media Technologies, Hamburg, Germany) software.

### Magnetoencephalography Recording

The whole experimental paradigm was composed of three behavioral test sessions after six MEG recording sessions (Figure 1B). Each MEG session included 100 sequences consisting of 30 sequences per condition and 10 staccato sequences. In individual staccato sequences, a staccato chord of 37.5-ms duration was presented in the third, fourth, or fifth chord. The participants were asked to detect staccato chords and to respond using a mouse. The response for staccato sequences was excluded in the MEG data analysis. In each behavioral session after MEG recording, 12 sequences per condition were randomly presented. For the conditions of *T*, *SM*, and *ST*, all participants were asked to identify each condition using the 1, 2, and 3 buttons on the keypad, respectively, instead of using chord labels of *T*, *SM*, and *ST*. The participants were instructed to distinguish between the three stimuli and to understand each task of the MEG/behavioral experiment. This process was done through training for a certain period of time before starting the main experiment. The musical stimuli were presented at the sound pressure level of 65 dB into MEG-compatible tubal insert earphones (Tip-300, Nicolet, Madison, WI, United States) using the STIM^2^ (Neuroscan, Charlotte, NC, United States) system. The whole experiment took about 2 h. MEG signals were recorded in a magnetically shielded room using a 306-channel whole-head MEG System (Elekta NeuroMag VectorView^TM^, Helsinki, Finland), with a sampling rate of 600.615 Hz using 0.1- to 200-Hz band-pass filter. Electrooculograms (EOGs) and electrocardiograms (ECGs) were simultaneously recorded to later remove ocular and cardiac noise.

### Magnetoencephalography Analysis

The environmental magnetic noise of raw MEG signals was eliminated by the temporal Signal-Space Separation (tSSS) algorithm in MaxFilter 2.1.13 (Elekta Neuromag Oy, Helsinki, Finland) (Taulu and Simola, 2006; Taulu and Hari, 2009). The 204 orthogonal planar gradiometer in 102 locations was used in the further analysis procedure. Source analysis of four regions of interest (ROIs) [bilateral IFGs and superior temporal gyrus (STGs)] was performed using BESA 5.1.8.10 (MEGIS Software GmbH, Gräfelfing, Germany). Multiple equivalent current dipoles (ECDs) for the bilateral IFGs and STGs were fit on the generators of P2m and ERANm (as magnetic counterparts of the P2 and ERAN, respectively), as estimated with the same procedures as in our previous studies (Kim et al., 2011, 2014). After the ECDs of P2m were estimated in the peak latency of 180–190 ms for an average of all in-key chords, the ECDs of ERANm were estimated in 140–220 ms for all ending chords (mean of the tonic, submediant, and supertonic chords). The ECDs were localized on the bilateral STGs involving auditory cortices for the P2m and on the bilateral IFGs for the ERANm. The multiple dipoles were more than 80% of the goodness of fit (GOF). The estimated dipoles in the IFG were superior and anterior to those in the STG (Maess et al., 2001; Kim et al., 2011, 2014). The x, y, and z in Talairach coordinates (millimeters) were −45.1, −8.9, and 1.9 in the left STG; 43.1, −2.6, and 2 in the right STG; −40.8, 18.5, and 15.6 in the left IFG; and 37.6, 21.2, and 15.1 in the right IFG, respectively (Supplementary Figure 1A). The signal for ECDs was extracted in 400-ms epochs after the onset of the ending chord using a 1- to 20-Hz band-pass filter for each participant. The 400 ms was the time window involving the peak latencies of P2m and ERANm in our previous studies (Kim et al., 2011, 2014; Supplementary Figure 1B).

Using the ECD signals of 400 ms in the multiple dipoles of the bilateral IFGs and STGs, we estimated the information flows in 12 directional connections between the bilateral IFGs and STGs for the three conditions. Effective connectivity for 12 connections was calculated by LTDMI (Jin et al., 2010). The LTDMI is an information-theoretic measure of functional coupling based on mutual information (MI) (Jin et al., 2011, 2012; Kim et al., 2020), which predicts information transmission between two time series.

Mutual information is defined as the quantity of information shared in two time series of *X*(*n*) and *Y*(*n*) (*n* = 1, 2, …, N), at N discrete points. The probability density function (PDF) of *X*(*n*) and *Y*(*n*) is *p*(*X*(*n*),κ)≡*p*(*X*(*n*)) and *p*(*Y*(*n*),κ)≡*p*(*Y*(*n*)) with *n* = 1, 2, …, bin, respectively. The MI is computed by *p*(*X*(*n*),*Y*(*n*)), the joint PDF between *X*(*n*) and *Y*(*n*), as follows:

(1) $$MI=MI_{XY}=MI_{YX}=MI\left(X\left(n\right),Y\left(n\right)\right)=-\sum_{k}p\left(X\left(n\right),Y\left(n\right)\right)\text{log}\frac{p\left(X\left(n\right),Y\left(n\right)\right)}{p\left(X\left(n\right)\right)p\left(Y\left(n\right)\right)}$$

If *X*(*n*) and *Y*(*n*) are completely identical, the MI is maximum. However, if two time series are independent of each other, the MI is zero. The directional information transmission between the two time series can be calculated by time-delayed MI (TDMI):

(2) $$\begin{array}{c} TDMI_{XY}=TDMI\left(X\left(n\right),Y\left(n+\tau \right)\right)=-\sum_{k}p\left(X\left(n\right),Y\left(n+\tau \right)\right)\text{log}\frac{p\left(X\left(n\right),Y\left(n+\tau \right)\right)}{p\left(X\left(n\right)\right)p\left(Y\left(n+\tau \right)\right)}\\ TDMI_{YX}=TDMI\left(Y\left(n\right),X\left(n+\tau \right)\right)=-\sum_{k}p\left(Y\left(n\right),X\left(n+\tau \right)\right)\text{log}\frac{p\left(Y\left(n\right),X\left(n+\tau \right)\right)}{p\left(Y\left(n\right)\right)p\left(X\left(n+\tau \right)\right)} \end{array}$$

Time-delayed MI can detect linear and non-linear correlations between two time series. Since the data length used in the present study (400-ms epoch) was insufficient to reconstruct a reliable PDF for general TDMI presented in Equation (2), we used LTDMI as an effective connectivity measure in this study. LTDMI is adopted as follows:

(3) $$\begin{array}{c} LTDMI_{XY}=LTDMI\left(X\left(n\right),Y\left(n+\tau \right)\right)=-\frac{1}{2}\log\left(1-\rho_{X\left(n\right)Y\left(n+\tau \right)}^{2}\right)\\ LTDMI_{YX}=LTDMI\left(Y\left(n\right),X\left(n+\tau \right)\right)=-\frac{1}{2}\text{log}\left(1-\rho_{Y\left(n\right)X\left(n+\tau \right)}^{2}\right) \end{array}$$

where, ρ*X*(*n*)*Y*(*n* + τ) and ρ*Y*(*n*)*X*(*n* + τ) are a cross-correlation coefficient. The τ of delay time was 120 ms. To estimate the linearized information flow between the time series, each time series is assumed with the Gaussian distribution function with zero mean, and variance $\sigma_{X}^{2}$, $\sigma_{Y}^{2}$, i.e., $p\left(X\right)=\frac{1}{\sqrt{2\pi \sigma^{2}}}\exp\left(-x^{2}/2\sigma^{2}\right)$. The LTDMI values were averaged over delay time. When the difference between *LTDMI*_*XY*_ and *LTDMI*_*YX*_ is positive, the information flow between *X* and *Y* was interpreted as “*X* to *Y*.”

Differences in the LTDMI values among the three conditions of *T*, *SM*, and *ST* in 12 connections were tested by the two-way repeated-measures analysis of variance (ANOVA). In all *post hoc* analysis steps, the alpha levels for multiple comparisons were adjusted by the Benjamin–Hochberg FDR correction (*p* < 0.05). Additionally, the group difference for the LTDMI values was tested by the independent *t*-test (*p* < 0.05).

In the MEG experiment, the mean CR for staccato chord detection was calculated for each participant. In the behavioral experiment, the difference between the three conditions for the CR was determined by the one-way repeated-measures for all conditions.

Correlation analysis was performed to test the relationships between the LTDMI value and CR for all conditions of all participants (i.e., for the merged data set of three conditions of 18 participants). In the correlation analysis for the LTDMI value and the CR, the correlation was tested using Spearman’s rank correlation because the data were not normally distributed. The correlation was calculated using the one-tailed test because the ambiguous stimuli lead to slower and less accurate responses than the easy stimuli (Sabri et al., 2006; Jastorff et al., 2009; Fleming et al., 2010). The alpha level was adjusted by the FDR correction for the multiple comparisons testing of the three conditions (*p* < 0.05). The Greenhouse–Geisser correction was applied because the sphericity of the data was violated *via* Mauchly’s sphericity test. All statistical analyses were performed using SPSS 21.0 software (IBM, Armonk, NY, United States).

## Results

### Linearized Time-Delayed Mutual Information Values for Three Conditions

**FIGURE 2:**
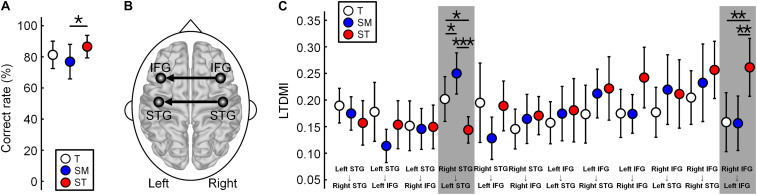
Difference in the linearized time-delayed mutual information (LTDMI) values for the three conditions. **(A)** Correct rate (CR) of *SM* is significantly lower than the CR of *ST*. The other pairs are not statistically significant. *p < 0.05. **(B)** Difference between the conditions for LTDMI was revealed in only two interhemispheric connections, which were termed “IFG-LTDMI” and “STG-LTDMI.” **(C)** The STG-LTDMIs were different between all pairs. In the IFG-LTDMI, the *SM* was higher in the other conditions. *p < 0.05, **p < 0.01, and ***p < 0.001 (FDR corrected). Error bars denote 95% confidence intervals. See also Supplementary Table 1. LTDMI, linearized time-delayed mutual information; CR, correct rate; IFG, inferior frontal gyrus; STG, superior temporal gyrus; FDR, false discovery rate.

The LTDMIs were calculated for 12 connections among four ROIs of the bilateral IFGs and STGs for three conditions of the *T*, *SM*, and *ST* in 19 participants. For the LTDMI values, we performed a two-way repeated-measures ANOVA with two factors of Condition and Connection. The ANOVA (*n* = 19) showed a significant main effect of Condition [*F*(1.872, 404.247) = 3.108, *p* = 0.049] and a significant interaction of Condition × Connection [*F*(20.587, 404.247) = 2.555, *p* = 0.0002], and a significant effect of Connection [*F*(1, 216) = 1.920, *p* = 0.038]. *Post hoc* one-way repeated-measures ANOVAs with the Condition factor in 12 connections confirmed a connection reflecting the difference among the three conditions. The difference between the three conditions was revealed only in two connections from the right to the left IFG [*F*(2, 36) = 6.526, *p* = 0.024, and FDR corrected] and from the right to the left STG [*F*(2, 36) = 12.373, *p* < 0.001, and FDR corrected] among 12 connections (Figures 2B,C; see also Supplementary Table 1).

Hereafter, we use the term “IFG-LTDMI” to refer to the LTDMI values from the right to the left IFG and the term “STG-LTDMI” to refer to those from the right to the left STG. In the two interhemispheric connections, the *SM* and *ST* of the most ambiguous and irregular conditions showed the highest STG-LTDMI and IFG-LTDMI, respectively.

In a *post hoc* paired *t*-test, the IFG-LTDMI (MEAN[SD] for IFG-LTDMI values of three conditions, *T* = 0.1584[0.1207], *SM* = 0.1563[0.1116], and *ST* = 0.2614[0.1186]) was higher for the *ST* than for the *T* [*t*(18) = 3.091, *p* = 0.009, and FDR corrected] and for the *SM* [*t*(18) = 3.223, *p* = 0.009, and FDR corrected], while the IFG-LTDMI for the *T* and *SM* was not significantly different [*t*(18) = 0.061, *p* = 0.952, and FDR corrected]. The STG-LTDMI (MEAN[SD] for STG-LTDMI values of three conditions, *T* = 0.2019[0.0919], *SM* = 0.2500[0.0843], and *ST* = 0.1438[0.0541]) was higher for the *SM* than for the *T* [*t*(18) = 2.691, *p* = 0.023, and FDR corrected] and the *ST* [*t*(18) = 5.357, *p* = 0.0001, and FDR corrected], while it was significantly higher for the *T* than for the *ST* [*t*(18) = 2.259, *p* = 0.037, and FDR corrected].

### Behavioral Response

During the MEG experiment, participants were asked to listen to each condition carefully and to detect the sequences including a staccato chord in order to check the level of attending to the condition (Figure 1B). All participants detected the staccato chord with more than 95% including the number of missed buttons. This indicates that the participants paid attention to musical stimuli. After the MEG experiment, participants performed a behavioral test discriminating among the three conditions (Figure 1B). The mean CR (*n* = 18) was lower in the *SM* (77.0%) than in the *T* (82.4%) and the *ST* (88.7%). The one-way repeated-measures ANOVA (*n* = 18, excluded one outlier) showed a significant main effect of Condition [*F*(2,34) = 4.799, *p* = 0.015]. In a *post hoc* analysis, the difference between the CRs was significant only between the *SM* and the *ST* (Figure 2A). The *SM* was significantly lower than the *ST* [*t*(17) = −2.574, *p* = 0.020]. There were no significant differences in the pairs of *T* vs. *SM* [*t*(17) = 1.772, *p* = 0.094] and *T* vs. *ST* [*t*(17) = −1.753, *p* = 0.098]. Additionally, in the questionnaire after the experiment on the participants, of the total participants, 15 responded to the questionnaire; among them, 10 responded “not certain” with the *SM*, while 13 responded “certain” for the *ST*.

### Correlation Between the Linearized Time-Delayed Mutual Information Values and Correct Rate

**FIGURE 3:**
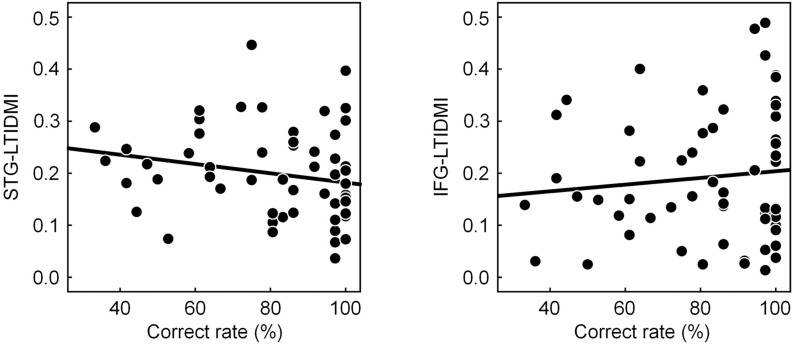
Correlation between correct rate (CR) and STG-LTDMI/IFG-LTDMI. The CR was only negatively correlated with the STG-LTDMI (*n* = 57, one-tailed Spearman’s rank correlation, Spearman’s ρ = −0.260, and *p* = 0.026). However, for the IFG-LTDMI, a significant correlation was not observed in both all participants, and subgroups. Best-fit trend lines for all participants (black solid line) are depicted in each graph. CR, correct rate; STG, superior temporal gyrus; LTDMI, linearized time-delayed mutual information; IFG, inferior frontal gyrus.

To confirm whether the STG-LTDMI reflected perceptual ambiguity, and, if it was so, whether it was specific for the STG-LTDMI among the IFG-LTDMI and the STG-LTDMI, we tested the correlation between the LTDMI values of STG-LTDMI/IFG-LTDMI and the behavioral response of CR. The correlation was tested using the values for all conditions and participants (*n* = 57, 3 conditions × 19 participants). A significant correlation with the CR was observed not in the IFG-LTDMI but in the STG-LTDMI (one-tailed Spearman’s rank correlation; STG-LTDMI, Spearman’s ρ = −0.260, *p* = 0.026; IFG-LTDMI, Spearman’s ρ = 0.064, and *p* = 0.319) (Figure 3).

## Discussion

The IFG-LTDMI was enhanced for the *ST* of the most irregular condition. The STG-LTDMI was enhanced for the *SM* of the most ambiguous condition. The processing of syntactic irregularity and perceptual ambiguity in the three conditions was dissociated in the IFG-LTDMI, and the STG-LTDMI, respectively. This implies that the brain interprets the three conditions as both “from regular to irregular” and “from ambiguous to unambiguous” conditions simultaneously.

The highest IFG-LTDMI for the *ST* is a further extension of the highest ERAN response elicited only for the most irregular condition (*ST*) in our previous study (Kim et al., 2011). The IFG-LTDMI in terms of effective connectivity may underlie the ERAN. Moreover, our data on IFG-LTDMI clearly show how both hemispheres of IFG are closely related to the processing of musical syntax. Also, it clearly shows what kind of connection between these areas is interpreted from the information introduced from the outside. Thus, the IFG-LTDMI from the right to the left IFGs is one step forward from the previous reports that the bilateral IFGs are the neural generators of ERAN (Maess et al., 2001; Sammler et al., 2009; Kim et al., 2011, 2014). Our data suggest that the left IFG and the right IFG interrelate in the processing of musical syntax, and in terms of effective connectivity.

The STG-LTDMI was the highest for the most ambiguous *SM*. The patterns of STG-LTDMI between all conditions were consistent with our hypotheses that the *SM* would be most ambiguous. In a language study, acoustic–phonetic processing is related to the STG (Callan et al., 2004). Auditory areas involving Heschl’s gyrus are activated by ambiguous phonemes (Kilian-Hutten et al., 2011). The STG is related to the processing of pitch and melody (Patterson et al., 2002; Schneider et al., 2005). The activity in the bilateral STGs is asymmetric in the right hemisphere (Patterson et al., 2002; Warrier and Zatorre, 2004; Bidelman and Grall, 2014). The right STG is more highly activated for deviant tones (Sabri et al., 2006) and is more sensitive to pitch congruency (Nan and Friederici, 2013) than the left STG. In contrast, the fundamental pitch in an ambiguous tone is processed in the left STG, while spectral pitch is processed in the right STG (Schneider et al., 2005). In a tone interval discrimination task, the STG dissociates difficult, and easy tasks (Sabri et al., 2006). Unambiguous stimuli of speech and ambiguous stimuli of speech like song differently activate the STG (Tierney et al., 2013). As the aforementioned studies, we interpret our findings in the bilateral STGs as indicating neural substrates for the perception of ambiguities implied in musical chord stimuli. In terms of voice leading, the *ST* of the most irregular condition with a salient melodic line (Kim et al., 2014) would be more distinguishable than other chords. However, since the *SM* shares the same voice leading as the *T*, this might have been another factor that could affect perceptual ambiguity. The sensory novelty and frequency of occurrence of the ending chords due to the distance between the two repeated chords varying between the three conditions might be a confounding factor affecting perceptual ambiguity (Koelsch et al., 2007; Zhou et al., 2019). Unlike the *T* of the major triad, also, the similar sonic quality of the minor triad may cause perceptual ambiguity between the *SM* and the *ST*. Therefore, the levels of perceptual ambiguity reflected in interhemispheric connectivity between the bilateral STG of STG-LTDMI might reflect the processes of all factors that could be heard in the chord stimulus (see also Supplementary Figure 2).

In previous studies, the connection between the IFG and STG is involved in the processing of syntax in music and language (Sakai et al., 2002; Sammler et al., 2009; Friederici, 2011; Musso et al., 2015). Moreover, an fMRI study using a real musical piece reported that the different levels of syntactic irregularity were reflected in functional connectivity between the IFG and STG (Seger et al., 2013). In another fMRI study on acoustic–phonetic processing, IFG–STG coupling is increased by ambiguous acoustic signal (Leitman et al., 2010). However, our data did not show the connection between the IFG and the STG in either syntactic irregularity or perceptual ambiguity. Instead, the connectivity was dissociated in IFG and STG. Furthermore, the direction of effective connectivity was from the right to left hemisphere in both the IFG-LTDMI and STG-LTDMI. We interpret dissociation of the IFG and STG in connectivity as indicating the functional segregation related to syntax, and ambiguity processing. Also, we interpret that the same direction of information transmission in the bilateral IFGs and STGs as indicating the different roles of bilateral hemispheres in music processing and indicating the IFG-LTDMI and STG-LTDMI commonly based on the rightward asymmetry.

Considering the STG-LTDMI, the *SM* is the most ambiguous among the three conditions, and the *ST* is less ambiguous than the *T*. The more ambiguous condition may be more difficult to discriminate than the less ambiguous condition. However, although the CR was only significantly different between the *SM* and the *ST*, the level of perceptual ambiguity was not perfectly confirmed in terms of behavioral results. Our data show that the participants could discriminate among the three chord sequences with reasonable accuracy (CRs for all conditions > 77%). We infer that the CR was overall high (ceiling effect), so perceptual ambiguity was probably not reflected in the CR. Therefore, we expected that the STG-LTDMI, an index reflecting perceptual ambiguity, would be related to the CR because perceptual ambiguity was involved in the CR, and the result was as expected. Most notable is that the significant correlation was only observed in the STG-LTDMI, not in the IFG-LTDMI. From these results, it can be inferred that the processing of perceptually ambiguous stimuli triggered the enhancement of connectivity in the STG-LTDMI.

The STG-LTDMI was the lowest for the *ST*. Which of the factors made each condition sound more ambiguous and less ambiguous cannot be clearly verified through the present results. However, it could be confirmed that at least the *SM*, which was likely to be confused with the other two conditions, was the most ambiguous; and the *ST*, the most irregular condition with a salient melodic line, was the most unambiguous. Syntactic error of the *ST* is well recognized by both musicians and non-musicians (Koelsch et al., 2000, 2001; Maess et al., 2001), and it can be inferred that the fact that more than half of the participants were non-musicians who were known to use chord identification strategies for melodic line (Sears et al., 2014) would all have influenced the present findings. From the fact that our findings were observed in the results including musicians and non-musicians, we could presume that discriminating, and perceiving the properties of chords could be a basic ability implicitly acquired in musical experiences just as it could detect syntactic violation (Koelsch et al., 2000).

## Limitation

Although our data suggest perceptual segregation of syntactic irregularity and perceptual ambiguity in chord sequences, and our data only focused on the four ROIs of the bilateral IFGs and STGs based on our hypothesis. This syntactic irregularity and perceptual ambiguity processes would be also examined in terms of the whole-brain analysis including the four ROIs of the bilateral IFGs and STGs on our hypothesis. Furthermore, interhemispheric connectivity might be more clearly revealed since the present study was only conducted on female participants. Which hemisphere of bilateral hemispheres is more dominant, or whether males can show different information flow in the directional connections, should be discussed and compared in future studies. Additionally, the main goal of the present study was not to select the factors affecting perceptual ambiguity and to identify the weight of each but to suggest the neurocognitive basis for the existence of perceptual ambiguity. Which of the aforementioned factors is perceptually dominant would be also addressed in future studies.

## Conclusion

To conclude, our findings demonstrate for the first time through neurobiological data that two processes, syntactic irregularity and perceptual ambiguity, coexist at the level corresponding to each condition in the musical stimuli of the chord sequence testing syntactic irregularity. The connectivity from the left to the right hemisphere in the IFG and the STG enhanced when the levels of the two processes were high, respectively. These results indicate that the two interhemispheric connectivities observed simultaneously in the IFG and the STG are neural substrates corresponding to the two processes.

## Data Availability Statement

The raw data supporting the conclusions of this article will be made available by the authors, without undue reservation.

## Ethics Statement

The studies involving human participants were reviewed and approved by Institutional Review Board of the Clinical Research Institute, Seoul National University Hospital (H-1001-020-306). The patients/participants provided their written informed consent to participate in this study. Written informed consent was obtained from the individual(s) for the publication of any potentially identifiable images or data included in this article.

## Author Contributions

CK and CC conceived the study and wrote the manuscript. SJ and JK contributed to analytic tools. YK and SY contributed to music-theoretical background. CK analyzed the data. All authors discussed the results and reviewed the manuscript.

## Conflict of Interest

The authors declare that the research was conducted in the absence of any commercial or financial relationships that could be construed as a potential conflict of interest.

## Publisher’s Note

All claims expressed in this article are solely those of the authors and do not necessarily represent those of their affiliated organizations, or those of the publisher, the editors and the reviewers. Any product that may be evaluated in this article, or claim that may be made by its manufacturer, is not guaranteed or endorsed by the publisher.
